# Brachial Neuritis
1Dr. C. W. Buckley: The Lancet, April 16, 1904.


**Published:** 1904-06-25

**Authors:** 


### BRACHIAL NEURITIS.[1]

Brachial neuritis may be compared to sciatica, and is equally painful and disabling. Like many other nervous diseases, it appears to be becoming more common. The prominent symptom is pain, sometimes of sudden onset, but more often occasional on making certain movements, and then gradually increasing and taking on a paroxysmal character; it may be referred to the region of the scapula, the shoulder, or the wrist, and may or may not affect the hand. The commonest form of brachial neuritis is that due to injury, as in catching at some object in order to break a fall, and here the appearance of the symptoms may be delayed for a week or more. In addition to pain there are local tenderness over the affected nerves, limitation of movement, and muscular wasting and paralysis in a greater or less degree. There may also be changes in and around the joint, giving rise to crackling noises on passive movements, and in this and other respects simulating the character of rheumatoid arthritis. Next to injury, gout is the most prominent factor in the etiology of brachial neuritis. In these instances the onset is sudden, with generalised pain and tenderness, often a sense of chill, and slight pyrexia. Probably most cases described as "rheumatic" are to be explained as the result of chill in a gouty subject. Some instances of brachial neuritis are reflex in origin, caused, for example, by a thoracic aneurism; in others, a lipoma in or about the scapular region has proved the primary source of irritation. In one case lead poisoning appeared to be the cause. The prognosis must take note of the fact that the condition rarely lasts less than three to four months, and that often it extends over a year; the older the patient, the more obstinate is the disease. The treatment, when the condition commences acutely, demands rest in bed, with purgatives and colchicum and alkalies in the gouty, salicylates in the rheumatic; aspirin with or without phenacetin is of distinct value. Locally, heat or cold, according to the comfort of the patient, should be employed, and mesotan is often very useful. The coal-tar analgæsics, and sometimes morphine, may be needed to control pain. When the acute stage has subsided, or in cases chronic from the outset, rest in a sling or with the arm bandaged to the side is still necessary. Alkalies, with potassium iodide or chloride of ammonium, may be given internally. No measure for the relief of pain is better than the free use of blisters, and galvanism has a valuable anodyne effect. Other forms of electricity are chiefly of use when neuralgia persists as a habit after the nerve lesion has been cured. Massage may be used to get rid of inflammatory effusion into nerve trunks and around joints, but should not be ordered so long as there is active inflammation. Bath and Buxton are suitable for balneological and climatological treatment.

[1] Dr. C. W. Buckley: The Lancet, April 16, 1904.

